# Alternaria toxins as casein kinase 2 inhibitors and possible consequences for estrogenicity: a hybrid in silico/in vitro study

**DOI:** 10.1007/s00204-020-02746-x

**Published:** 2020-04-23

**Authors:** Georg Aichinger, Luca Dellafiora, Foteini Pantazi, Giorgia Del Favero, Gianni Galaverna, Chiara Dall’Asta, Doris Marko

**Affiliations:** 1 Department of Food Chemistry and Toxicology, Faculty of Chemistry, University of Vienna, Austria. Währinger Str. 42, 1090 Vienna, Austria; 2 Department of Food and Drug, University of Parma, Area Parco delle Scienze 27/A, 43124 Parma, Italy

**Keywords:** Alternariol, Altertoxin II, Estrogen receptor, Mycotoxin, Estrogen receptor

## Abstract

Emerging mycotoxins produced by *Alternaria* spp. were previously reported to exert cytotoxic, genotoxic, but also estrogenic effects in human cells. The involved mechanisms are very complex and not fully elucidated yet. Thus, we followed an in silico target fishing approach to extend knowledge on the possible biological targets underlying the activity of alternariol, taken as the signature compound of *Alternaria* toxins. Combining ligand-based screening and structure-based modeling, the ubiquitous casein kinase 2 (CK2) was identified as a potential target for the compound. This result was validated in a cell-free in vitro CK2 activity assay, where alternariol inhibited CK2 with an IC_50_ of 707 nM. As CK2 was recently discussed to influence estrogen receptor (ER) transcription and DNA-binding affinity, we assessed a potential impact on the mRNA levels of ERα or ERβ by qRT-PCR and on nuclear localization of the receptors by confocal microscopy, using estrogen-sensitive Ishikawa cells as a model. While AOH did not affect the transcription of ERα or ERβ, an increase in nuclear localization of ERα after incubation with 10 µM AOH was observed. However, this effect might be due to ER binding affinity and therefore estrogenicity of AOH. Furthermore, in silico docking simulation revealed not only AOH, but also a number of other *Alternaria* toxins as potential inhibitors of CK2, including alternariol monomethyl ether and the perylene quinone derivative altertoxin II (ATX-II). These findings were representatively confirmed in vitro for the perylene quinone derivative altertoxin II, which was found to inhibit the kinase with an IC_50_ of 5.1 µM. Taken together, we propose CK2 inhibition as an additional mechanism to consider in future studies for alternariol and several other *Alternaria* toxins.

## Introduction

Fungi of the *Alternaria* genus occur ubiquitously and grow under a wide range of conditions. They can infest crops designated for human food production and thereby their toxic secondary metabolites can be found in feed and food. *Alternaria* toxins are of high interest in toxicology and belong to the so-called “emerging mycotoxins”, a term introduced for mold metabolites which exert toxic effects but are not regulated yet by authorities, due to still insufficient data on toxicity and/or occurrence. The high chemical diversity among the produced toxins results in a complex toxicological profile of *Alternaria* contaminations, which is still not entirely elucidated.

**Fig. 1:**
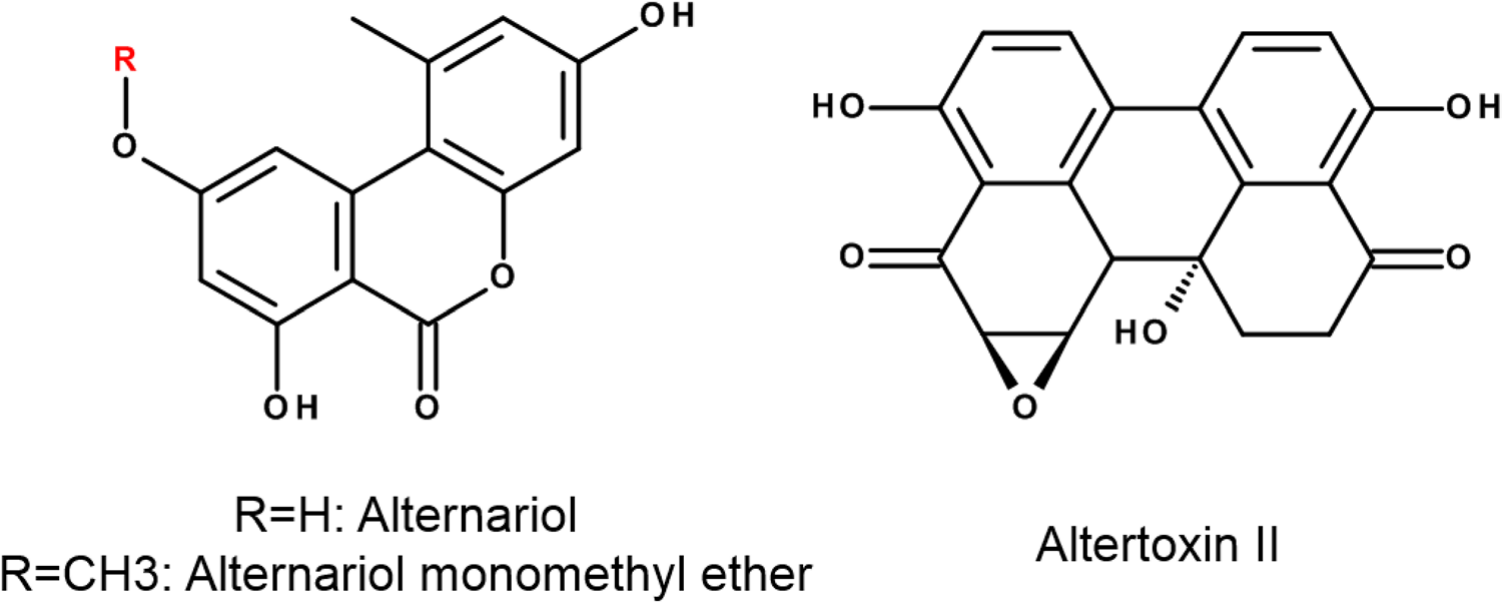
Chemical structures of alternariol, its monomethyl ether and altertoxin II

The composition of the produced toxin mixtures largely depends on both, the fungal strain and the growth conditions. In general, the most abundant metabolites of *Alternaria* spp. are tenuazonic acid, a substance with quite low and solely acute toxic properties, and alternariol (AOH, Fig. 1) (Zwickel et al. 2018). Regarding the toxicity of *Alternaria* contaminations, the latter is considered a lead compound, as it was repeatedly found in commercial food samples and was reported to exert a number of distinct adverse bioactivities (Ostry 2008; Puntscher et al. 2018b).

Together with its co-occurring monomethyl ether (AME), AOH was reported to act cytotoxic and genotoxic in human cells. For these effects, its ability to poison human topoisomerases, enzymes involved in the maintenance of DNA topology, appears to play a central role (Fehr et al. 2009). These DNA-damaging properties are currently considered as the main toxicological concern of *Alternaria* toxins by regulative authorities (EFSA 2016). Apart from that, AOH has recently been found to influence inflammatory responses (Kollarova et al. 2018; Solhaug et al. 2015).

Of particular novel interest are reports on the endocrine disruptive potential of the compound. AOH has been described as an agonist for human estrogen receptors (ER) (Lehmann et al. 2006) and the androgen receptor (Stypuła-Trębas et al. 2017). In addition, several of its metabolites were found to induce ER-dependent gene expression (Dellafiora et al. 2018b). Much attention has been given to the observation that despite the fact that AOH is only able to exert those effects at high concentrations, much lower doses are sufficient to potentiate the impact of other xenoestrogens like genistein or zearalenone, an effect whose mechanism is still unclear (Vejdovszky et al. 2017a, b).

In a recent study on rats, the systemic bioavailability of AOH and AME was described to be comparably low, with 6–10% of the compounds excreted via the urine, while 87% of the administered AME was found to remain in the feces (Puntscher et al. 2019). Martins et al. (2019) partly found considerable amounts (up to 24.6 µg/L) of AOH in some human urine samples collected during their biomonitoring approach to assess the exposure of the Portuguese population, which underlines the importance to gather more toxicological data for risk assessment of this mycotoxin.

Other, less studied *Alternaria* toxins include several compounds of the perylene quinone family. Some of those, e.g., altertoxin II (ATX-II, Fig. 1) or stemphyltoxin III, carry an epoxide moiety by which they might be able to react with different macromolecules, including the DNA. ATX-II has been shown to by far exceed the genotoxic potential of AOH and AME and to represent one of the main genotoxic compounds in extracts from *Alternaria alternata*-infested rice (Schwarz et al. 2012). However, until now ATX-II has not been detected in processed food, probably due to its high reactivity with other food constituents (Aichinger et al. 2018). Furthermore, the systemic bioavailability of epoxide-carrying perylene quinones was found to be very low in a recently published study on rats (Puntscher et al. 2019).

Altertoxin I (ATX-I), which does not possess a functional epoxy group, lacks the genotoxic impact of its relative, but was found in food samples as reported in some studies (Fraeyman et al. 2017). Several additional metabolites, e.g., altenusin, altenuene, iso-altenuene or altenuisol are produced by *Alternaria* spp. in considerably low concentrations (Zwickel et al. 2018) and although a few studies addressed potential adverse effects, they still need to be thoroughly characterized from a toxicological point of view.

Currently, scientific interest in *Alternaria* toxins is focused on measuring contamination levels in food, on assessing the impact of naturally occurring complex mixtures, but also on elucidating mechanisms by which they might influence cellular pathways. Thus, preceding this study, AOH was included in an in silico target fishing approach to screen for a number of targets, including casein kinase 2 (CK2). This protein is involved in numerous cell signaling pathways and also serves as a novel target for chemotherapy (Litchfield 2003; Rabalski et al. 2016). As a pleiotropic kinase, CK2 has been discussed to interact with the complex and not entirely elucidated processes contributing to estrogenic signaling pathways. ERs contain several phosphorylation sites with different functionalities and some of them could pose a substrate to CK2 (Maggi 2011). In particular, Williams et al. showed the kinase to phosphorylate ERα at Ser-282 and Ser-559 and thereby to inhibit a ligand-independent activation of the receptor. Of note, Ser-282 resides in the hinge region opposite to the DNA-binding domain of the receptor, which suggests a mode of action via a modulation of DNA-binding affinity or receptor dimerization (Williams et al. 2009). Furthermore, the activation of the p-p38/CK2 complex was reported to cause a downregulation of ER-α transcription, expression and thus activity (De Amicis et al. 2013), while CK2 inhibition was recently correlated with an increased ERα expression in breast cancer patients (Williams et al. 2015).

Thus, in the present study, we performed in silico target fishing, coupling ligand-based virtual screening and structure-based molecular modeling, and validated results in vitro to confirm the inhibitory activity of AOH towards the CK2. The survey was then expanded to other *Alternaria* toxins. Additionally, we tried to provide first insights of a possible influence of the observed inhibition on potential endocrine effects of AOH using Ishikawa cells and the enzyme alkaline phosphatase as a natural reporter for estrogenicity.

## Materials and methods

### Chemicals and assay kits

Cell culture media and supplements were purchased from GIBCO Invitrogen (Karlsruhe, Germany), cell culture flasks and dishes from Sarstedt (Nürnbrecht, Germany) and from Ibidi (Ibidi GmbH, Martinsried, Germany). 17β-Estradiol (E2), alternariol (AOH), quercetin (QUE) and Triton-X 100 were purchased from Sigma-Aldrich (Schnelldorf, Germany). CK2α1 Kinase Enzyme System, ADP-Glo^™^ Assay as well as Maxwell^™^ 16 LEV simplyRNA Cell Extraction kits were obtained from Promega (Mannheim, Germany). QuantiTect^®^ Reverse Transcription kit, QuantiTect^®^ SYBR^®^ Green PCR kit and primers for *ESR1*, *ESR2*, *ALPP*, *GADPH* and *ACTB* were purchased from Quiagen (Hilden, Germany). ATX-II was isolated from an extract of *Alternaria alternata* cultured on rice as previously described (Puntscher et al. 2018a).

### Cell culture

The human endometrial cancer cell line Ishikawa was purchased from ECACC (Wiltshire, UK). Cell stocks were prepared from the first passages after delivery, monitored for mycoplasma contaminations and stored in liquid nitrogen. Before starting experiments, cells were re-cultivated and grown in “growth medium”, a minimum essential medium (MEM) supplemented with 5% (v/v) heat-inactivated fetal bovine serum (FBS), 2 mM l-glutamine and 100 u/mL penicillin/streptomycin (P/S). For experiments, cells were seeded in “assay medium”, a Dulbecco’s Modified Eagle Medium/F-12 nutrient mixture (DMEM/F12) not containing phenol red but supplemented with 5% charcoal–dextran stripped (CD-) FBS and 100 u/mL P/S.

### Target fishing

The computational study relied on a target fishing protocol as previously reported (Dellafiora et al. 2019). In more detail, AOH was used as template in a ligand-based virtual screening querying a database of compounds with known activity and biological targets to identify hits with degrees of physico–chemical similarities to AOH. The ligands database was built using the ligands repository available in the RCSB PDB databank (https://www.rcsb.org) (Berman et al. 2000) that groups all the ligands bound to the proteins deposited in the databank. In particular, the non-redundant set of ligands was downloaded from the Ligand Expo Download page (https://ligand-expo.rcsb.org) in the 3D chemical table file format (sdf; 24,885 compounds; last database access in August, 23rd 2017), taking into account for the analysis only ligands with a molecular weight ranging between 200 and 500 g/mol (15,248 entries were selected). This subset of compounds was used for the ligand-based virtual screening using the FLAP software (https://www.moldiscovery.com) (Baroni et al. 2007). The default software setting was used to create the database and virtual screening was performed using the bit string mode to reduce the computation time.

### Structure-based molecular modeling

The structure-based modeling relied on pharmacophoric modeling, docking simulations and molecular dynamics. Pharmacophoric modeling of the CK2 binding site was performed using the Flapsite tool of the FLAP software (www.moldiscovery.com), while the GRID algorithm was used to investigate the corresponding pharmacophoric space (Baroni et al. 2007; Carosati et al. 2004). Docking simulations were done using the software GOLD (Genetic Optimization for Ligand Docking), as it previously succeeded in providing reliable architectures of binding and in predicting the activity of compounds of toxicological concern (e.g., Dellafiora et al. 2015; Maldonado-Rojas et al. 2011). Specifically, the model of the catalytic domain of human CK2 derives from the crystallographic structure having PDB code 3OWL (Prudent et al. 2010). Software setting, protocols and model and ligands preparation reported by Dellafiora et al. (2017) were used. As an exception, the use of external scoring functions was omitted as the GOLD’s internal scoring function GOLDScore succeeded in analyzing the reference set of compounds (vide infra). Additionally, since GOLD implements a genetic algorithm that may induce variability in the results, all analyses were done in quintuplicate to exclude non-causative scores assignment. Possible outliers were removed to a maximum of one for each run after being identified with the modified Thompson Tau test, as previously reported (Dellafiora et al. 2018a). In addition, molecules showing a coefficient of variation higher than 20% were considered unable to find a stable binding architecture. Accordingly, they were considered unable to favorably bind the pocket. Molecular dynamic (MD) simulation was done to investigate the permanence of AOH, AME and ATX-II into the ATP-binding site of CK2. For each compound, the binding pose calculated by docking simulations was used as input for MD using GROMACS (version 5.1.4) (Abraham et al. 2015) with CHARMM27 all-atom force field parameters support (Best et al. 2012). AOH, AME and ATX-II have been processed and parameterized with CHARMM27 all-atom force field using the SwissParam tool (https://www.swissparam.ch) (Zoete et al. 2011). Protein–ligand complexes were solvated with SPCE waters in a cubic periodic boundary condition and counterions (Na^+^ and Cl^−^) were added to neutralize the system. Prior to MD simulation, the systems were energetically minimized to avoid steric clashes and to correct improper geometries using the steepest descent algorithm with a maximum of 5000 steps. Afterwards, all the systems underwent isothermal (300 K, coupling time 2 psec) and isobaric (1 bar, coupling time 2 psec) 100 psec simulations before running 50 nsec simulations (300 K with a coupling time of 0.1 psec and 1 bar with a coupling time of 2.0 psec).

### CK2 activity assay

The inhibition of the activity of CK2 to phosphorylate casein was measured by assessing the corresponding conversion of ATP to ADP, using the coupled CK2α1 Kinase Enzyme System/ADP-Glo^™^ Assay kits from Promega (Mannheim, Germany) according to the manufacturer’s manual. Briefly, a white 96-well plate was used to incubate 2 ng/µL of CK2 for 60 min at 37 °C with different concentrations of AOH or ATX-II in kinase reaction buffer with a final concentration of 5% (v/v) DMSO, 100 µM ATP, 100 µM DTT and 0.2 mg/mL casein. 10 µM QUE was used as a positive control. The reaction was stopped by adding ADP-Glo reagent incubated for another 40 min at room temperature. Subsequently, the kinase reaction reagent was added and after 30 min of incubation at room temperature, the luminescence was captured with a BioTek^™^ Synergy H1 microplate reader.

### Quantitative real-time (qRT-) PCR

100,000 Ishikawa cells/well were seeded in 12-well plates and grown for 72 h in assay medium. Then, cells were incubated with the solvent control, different concentrations of AOH or 10 µM QUE for 24 h. RNA extraction was performed using a Maxwell^™^ 16 LEV simplyRNA kit according to the manufacturer’s manual. Briefly, the medium was removed, cells were washed with PBS, and a homogenization solution was added to the wells. Cells were harvested, singularized and lyzed. The lysates were transferred to cartridges and RNA extraction was carried out with the Maxwell^™^ 16 instrument. After centrifugation, the RNA content of the resulting supernatant was determined with a Thermo Scientific^™^ Nanodrop 2000/2000c spectral photometer. The QuantiTect1Reverse Transcription Kit was used to transcribe the RNA to cDNA according to the manufacturer’s protocol. In brief, 1 µg RNA of each sample was mixed with RNAse-free water plus a gDNA wipeout solution and incubated for 2 min at 42 °C. The RT-MasterMix was added and the tubes were incubated for another 15 min at 42 °C and for 2 min at 95 °C. The resulting solutions of cDNA were stored at − 20 °C until further processing.

qRT-PCR was conducted with primers for *ESR1*, *ESR2* and *ALPP* genes, with *GAPDH* and *ACTB* serving as housekeeping genes. cDNA was mixed with a SYBR green kit and the respective primer to a total volume of 20 µl and heating steps were performed with a StepOne Plus^™^ thermocycler following the manufacturer’s protocol. For analysis of obtained CT values, housekeeping genes were used to calculate 2^−ΔΔC^ values for relative quantification of gene transcription as suggested by Schmittgen and Livak (2008).

### Confocal microscopy

For the immunolocalization of ERα, Ishikawa cells were stained as previously described (Dellafiora et al. 2018b). Briefly, 40,000 cells/well were seeded in 8-well chamber microscopy slides (Ibidi GmbH, Martinsried, Germany) and incubated with AOH (0.1–10 µM) or a solvent control for 24 h. At the end of the incubation protocol, they were fixed in pre-warmed 3.7% formaldehyde and permeabilized with Triton X (0.2%) for 10 min. Unspecific binding sites were blocked with donkey serum (2%, 1 h) and ERα was stained using anti-ERα (D-12): sc-8005. Anti-Lamin B(c-20): sc-6216 antibody was used for the identification of the nucleus (both antibodies were used at dil. 1:250, Santa Cruz Biotechnology, Inc., Heidelberg Germany). Afterwards, cells were rinsed three times with washing buffer (0.01% Triton in PBS)and incubated with fluorescent labeled secondary antibodies, namely Alexa Fluor 647 Donkey Anti Goat (A-21447; Life Technologies, Thermo Fisher Scientific) and Alexa Fluor 488 Donkey Anti-Mouse (715-545-150, Jackson ImmunoResearch Laboratories, USA; 1 h 30 min dilution 1:500). At the end of the incubation, cells were washed additionally three times with washing buffer and three times with PBS. At the end of the staining, cells were mounted with Roti-Mount FluoCare (Roth, Graz, Austria) with DAPI to counterstain cell nuclei. If not otherwise specified, reagents were from Invitrogen by Thermofisher Scientific (Waltham US).

Images were acquired with a laser scanning confocal microscope LSM Zeiss 710 equipped with ELYRA PS.1 system with a Plan Apochromat 63X/1.4 oil objective and an AndoriXon 897 (EMCCD) camera. Data depicting the co-localization between ERα and the nuclear compartment (DAPI) were obtained with the software ZenZeiss 2012. Data were compared with the Mann–Whitney test and significance was attributed at threshold values < 0.05.

## Results

### Target fishing for AOH

A database of ligands with known activity and already described biological targets was screened using AOH as template to find hints with degrees of physico-chemical similarities. The rationale behind the use of this approach to identify possible unexpected targets of AOH relied on the principle that similar compounds may compete for the same biological targets. Accordingly, they may have a degree of analogy in initiating specific molecular events as well as in eliciting similar biological effects (McKinney et al. 2000). The Chemical Component Dictionary of the RCSB PDB repository (https://www.rcsb.org) (Berman et al. 2000) was used to generate the database of compounds analyzed with the ligand-based virtual screening (15,248 entries in total). The screening was done using the FLAP software (see material and method for more details) and the output sorted according to the FLAP “distance score”, which is an overall estimate of the divergence of compounds from the template (AOH in this case) in terms of physico–chemical properties (the lower the scores, the more similar the compounds). Only the top-scored ligands in the bound state with human proteins were taken into account for the analysis.

The three top-scored compounds were 11-chloro-8-methyl-7H-benzo[e]pyrido[4,3-b]indol-3-ol (PDB code 19E; distance score of 9.8), coumestrol (PDB code CUE; distance score 9.9) and ellagic acid (PDB code REF; distance score of 9.9). In more detail, 19E is a benzopyridoindole derivative inhibiting human CK2 via ATP-competitive mechanism (Prudent et al. 2010). Coumestrol is a natural polyphenol with a wealth of biological activities already described, including estrogenicity (Nwachukwu et al. 2017). Indeed, it was present in the PDB databank in the bound state with the human ERα. Ellagic acid is a polyphenolic compound abundant in many fruit and vegetables with numerous beneficial activity, including anti-inflammatory and antioxidant properties (Derosa et al. 2016). In the PDB databank, it was found in the bound state with human CK2 being an ATP-competitive inhibitor analogously to 19E (Sekiguchi et al. 2009). On the basis of these results, certain degrees of inhibitory activity of AOH against human CK2 were thought likely given the chemical analogies with known ATP-competitive inhibitors.

### Molecular modeling of AOH within CK2

The possible interaction of AOH within the ATP-binding site of human CK2 was thoroughly analyzed by means of a structure-based molecular modeling that relied on docking simulations, pharmacophore modeling and molecular dynamics.

**Fig. 2:**
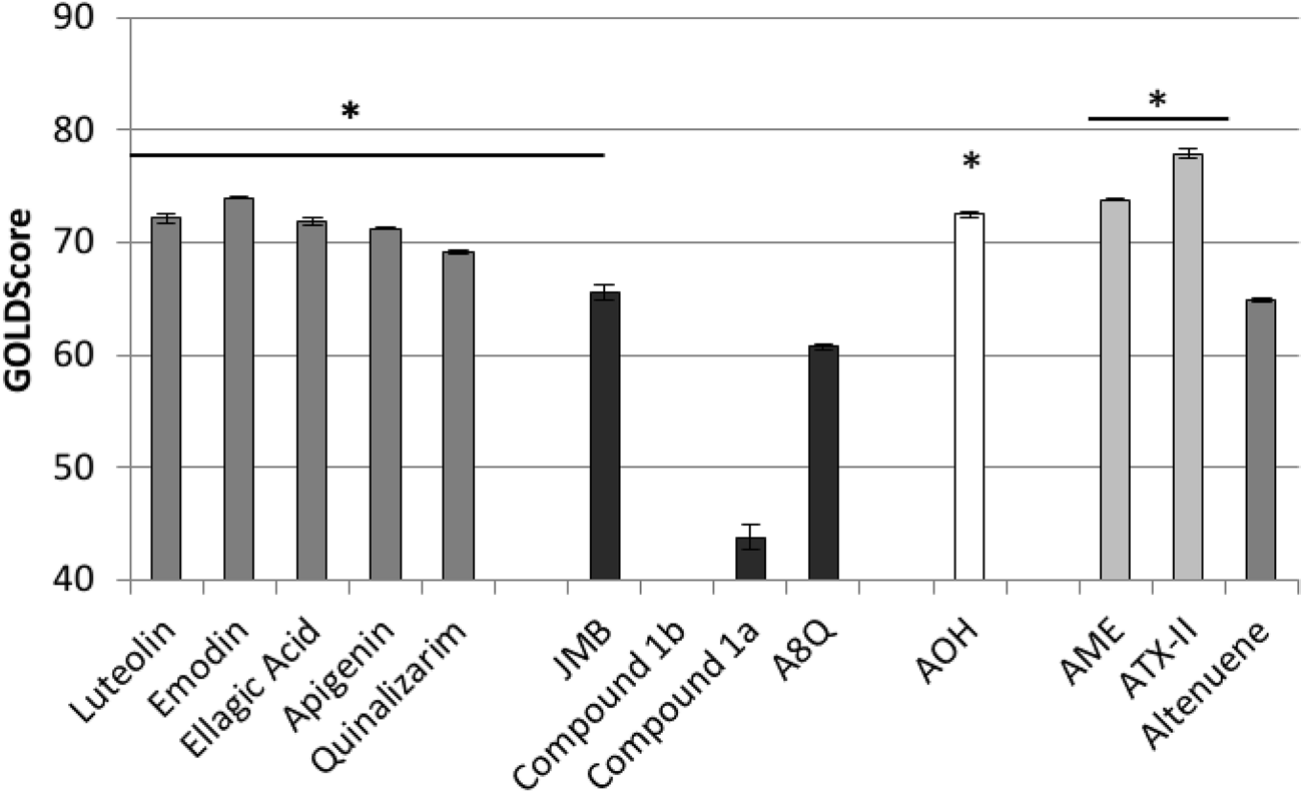
Docking scores of the validation set, AOH, AME, ATX-II and altenuene. Significance to negative controls (black bars): * < 0.01; according to Games-Howell post hoc test

Docking simulations aimed at assessing the capability of AOH to fit the ATP-binding pocket of CK2. They were done using the software GOLD (Genetic Optimization for Ligand Docking), as it previously succeeded in providing reliable architectures of binding and in predicting the activity of compounds of toxicological concern (e.g., Dellafiora et al. 2015; Maldonado-Rojas et al. 2011). Nevertheless, a fit-for-purpose validation study to assess the procedure reliability was done, according to previous studies (e.g., Dellafiora et al. 2015). To this end, the model was challenged with a validation set including both positive and negative controls retrieved from the literature. The set of positive controls included molecules with previously characterized CK2 inhibitory activity via ATP-competitive mechanism. The set of negative controls included instead molecules that were either unable to inhibit CK2 or that were unable to bind the ATP site of CK2 (Table 1). As shown in Fig. 2, all the ATP-competitive inhibitors recorded scores significantly higher than those recorded by negative controls (*p* < 0.01, according to Games-Howell post hoc test). In particular, the negative control W16 was considered unable to stably fit the pocket due to the low score (60.8 units) and high coefficient of variation (23%). On this basis, the procedure proved reliability in qualitatively predicting the inhibitory activity via ATP-competitive mechanism. Of note, computational scores may be proportional to the physico-chemical fitting of molecules within the protein pocket (the higher score, the higher the ligand-pocket match). In the case under analysis, two negative controls recorded relatively high scores reasonably due to a partial match with the pocket. Nonetheless, the statistical difference in respect to the positive controls proved the meaningfulness of the categorization drawn out. The model was than challenged with AOH recording a score significantly higher than those recorded by negative controls (*p* < 0.001 according to Games-Howell post hoc test) and comparable to those of ellagic acid and luteolin (*p* = 0.9 and *p* = 0.1, respectively, according to Games-Howell post hoc test) supporting its capability to dock the ATP pocket.

**Table 1:** List of compounds forming the set of positive and negative controls

Compounds	Inhibitory activity	References
Luteolin	Yes	Lolli et al. (2012)
Emodin	Yes	Sekiguchi et al. (2009)
Apigenin	Yes	Sekiguchi et al. (2009)
Ellagic acid	Yes	Lolli et al. (2012)
Quinalizarim	Yes	Papinutto et al. (2012)
JMB	No	De Fusco et al. (2017)
Benzothiazole derivative 1A	No	Prudent et al. (2008)
Benzothiazole derivative 1B	No	Prudent et al. (2008)
A8Q	No	Brear et al. (2018)
W16	No	Laudet et al. (2008)

**Fig. 3:**
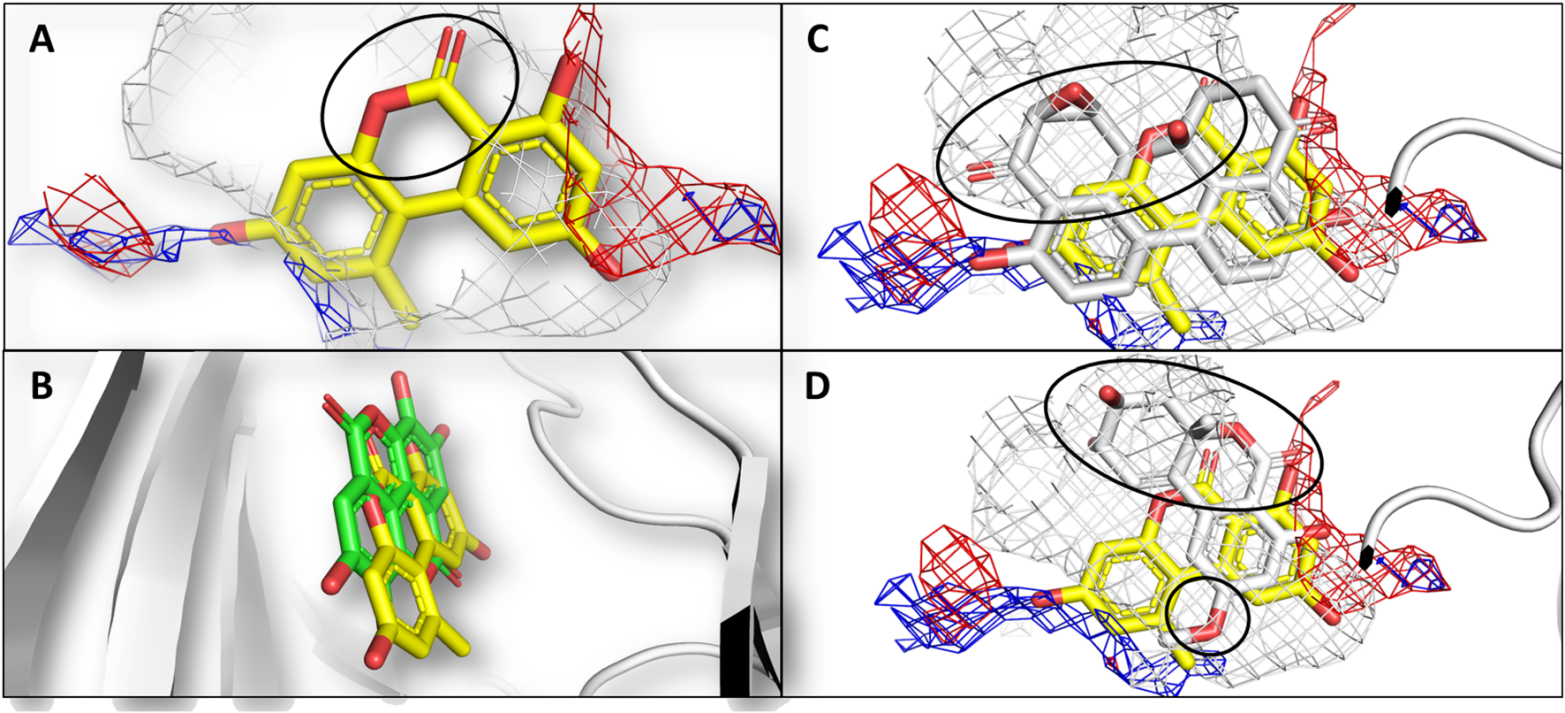
Calculated binding poses of AOH, ATX-II and altenuene. Protein is represented in white cartoon, while ligands are shown in sticks. Grey, red, and blue meshes indicate regions sterically and energetically favorable to receive hydrophobic, hydrogen bond acceptor, and hydrogen bond donor groups, respectively. Black rings indicate the improper arrangement of groups with respect to the pharmacophoric imagine of pocket. **a** Comparison between the calculated pose of AOH and the pharmacophoric imagine of pocket. **b** Calculated pose of AOH (yellow) superimposed to the crystallographic architecture of ellagic acid (green) (PDB structure 2ZJW) (Sekiguchi et al. 2009). **c** Calculated pose of ATX-II (white) superimposed to that of AOH (yellow). **d** Calculated pose of altenuene (white) superimposed to that of AOH (yellow) (color figure online)

The capability to fit the ATP site was further analyzed comparing the calculated binding pose with the pharmacophoric imagine of the space available for arranging ligands. As shown in Fig. 3, AOH well complied with the distribution of hydrophobic and hydrophilic space, except for the arrangement of the polar α-pyrone moiety into the hydrophobic region of the pocket. However, the calculated binding pose was in strong agreement with the crystallographic architecture of ellagic acid (Fig. 3b), which shows strong structural analogies with AOH, further supporting the plausibility of the calculated pose.

**Fig. 4:**
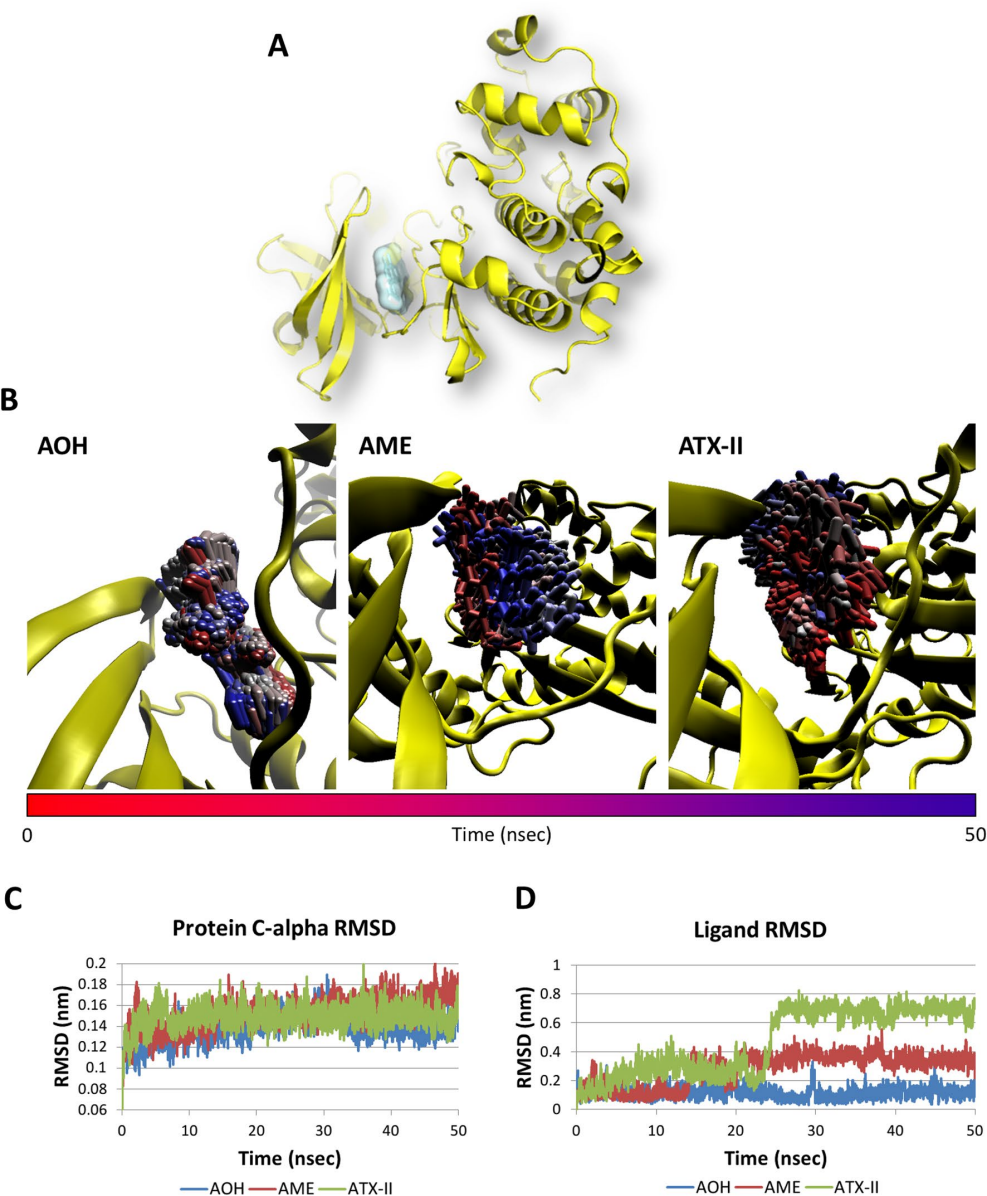
Results of molecular dynamic simulations. **a** Structure of the AOH–CK2 complex. The protein is represented in yellow cartoon, while AOH is represented in white surface. **b** Time-step representation of CK2 trajectory in complex with AOH, AME or ATX-II. The from-red-to-blue color switch indicates the stepwise changes of ligand coordinates along the MD simulation. **c** RMSD plot of protein C-alpha in complex with AOH, AME or ATX-II. **d** RMSD plot of AOH, AME or ATX-II (color figure online)

In addition, the capability of AOH to persist within the ATP pocket was assessed by means of molecular dynamic simulations. The root-mean-square deviation (RMSD) analysis of protein C-alpha and ligand atomic coordinates were analyzed to measure the structural stability of complex. The RMSD fluctuations of both, CK2 and the docked pose of AOH, were quite stable along the dynamic at the timescale under analysis, pointing to the overall geometrical stability of the complex. In addition, the time-step analysis of the AOH trajectory also revealed a stable interaction with the binding site confirming the capability to persist therein (Fig. 4c).

### Predicted CK2 inhibition of further *Alternaria* toxins

To identify additional *Alternaria* mycotoxins potentially able to inhibit CK2, the model was further challenged docking AME, ATX-II and altenuene. As shown in Fig. 2, AME and ATX-II recorded scores significantly higher than those recorded for negative controls (73.8 ± 01 and 77.9 ± 0.4, respectively; *p* < 0.01 according to Games-Howell post hoc test) and in the range of those recorded by positive controls. Conversely, the score of altenuene was significantly lower than those of positive controls (*p* < 0.01 according to Games-Howell post hoc test) and in the range of those recorded by negative controls (*p* = 0.6 to JMB, according to Games-Howell post hoc test). The inspection of the binding poses revealed that AME and ATX-II, but not altenuene, adopted architectures of binding mimicking the crystallographic pose of ellagic acid, as shown by AOH (Fig. 3). In addition, AME and ATX-II, but not altenuene, were found complying with the overall distribution of hydrophobic and polar space into the surface groove similarly to AOH. Nevertheless, ATX-II was found placing polar groups into hydrophobic region of the pocket to a larger extent than AOH (Fig. 3c). A pocket fitting worse than that of AOH could be hypothesized accordingly. Conversely, altenuene adopted an orientation markedly different than that of AOH and the arrangement of many groups did not comply with the pharmacophoric space of the pocket (Fig. 3d). On the basis of these results, AME and ATX-II, but not altenuene, were considered able to favorably dock the pocket.

Based on the promising docking results collected for AME and ATX-II, both compounds underwent molecular dynamic simulations to check their capability to persist within the ATP pocket over the time. As shown in Fig. 4, neither of the two molecules recorded geometries of interaction stable as that of AOH. In particular, while the CK2 structure was found geometrically stable in both complexes (Fig. 4c), both AME and ATX-II showed an early and marked RMSD increase (Fig. 4d) pointing to their lower capability compared to AOH to stably fit the ATP-binding site over the time. This evidence was confirmed by the visual inspection of molecule trajectories that revealed for AME and ATX-II an interaction with CK2 geometrically less stable than as compared to AOH (Fig. 4b). Indeed, AME and ATX-II showed an occupancy broadly spread over the ATP pocket that might lead to detachment earlier than AOH as time goes by. On the basis of these results, ATX-II and AME were judged to fit with the ATP pocket of CK2 to a lower extent than AOH, though their interaction can be expected leading to a certain degree of CK2 inhibition.

Taken together the results of docking analysis and molecular dynamic simulation, ATX-II was chosen along with AOH for the experimental trials given the highest docking score recorded among the *Alternaria* toxins under analysis.

### CK2 inhibition in vitro

**Fig. 5:**
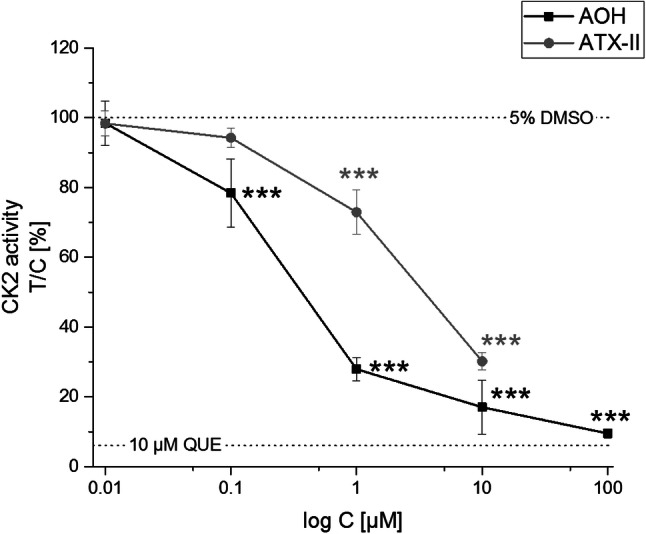
CK2 activity assay. Points indicate the concentration-dependent influence of AOH and ATX-II on the activity of CK2 in relation to the solvent control (5% v/v DMSO) ± SD of at least three independent experiments. The effect of each the solvent and the positive control (10 µM QUE) is displayed as a dotted line. Significant differences to the respective no-effect level were calculated by one-way ANOVA, followed by Fisher’s LSD post hoc testing, and are indicated by “*” (*p* < 0.05), “**” (*p* < 0.01) or “***” (*p* < 0.001)

The kinase activity assay is based on measuring the conversion of ATP to ADP during a 1 h reaction of recombinant CK2α1 with casein. Using this cell-free method, we found AOH to inhibit CK2 activity in a dose-dependent matter at concentrations ≥ 100 nM (Fig. 5). With micromolar concentrations applied, enzyme activity was reduced by up to 91% (for 100 µM of AOH). An IC_50_ value of 707 nM was calculated using the “DoseResp” curve fitting option in the Orgin2016G software. ATX-II also impaired CK2 functionality, albeit at higher concentrations. This inhibition was significant for ≥ 1 µM and the IC_50_ was calculated to reside at 5.1 µM. 10 µM of quercetin, which was previously reported to act as a CK2 inhibitor (Russo et al. 2017), served as a positive control and suppressed CK2 activity by 94%.

### ER and AlP gene transcription

**Fig. 6:**
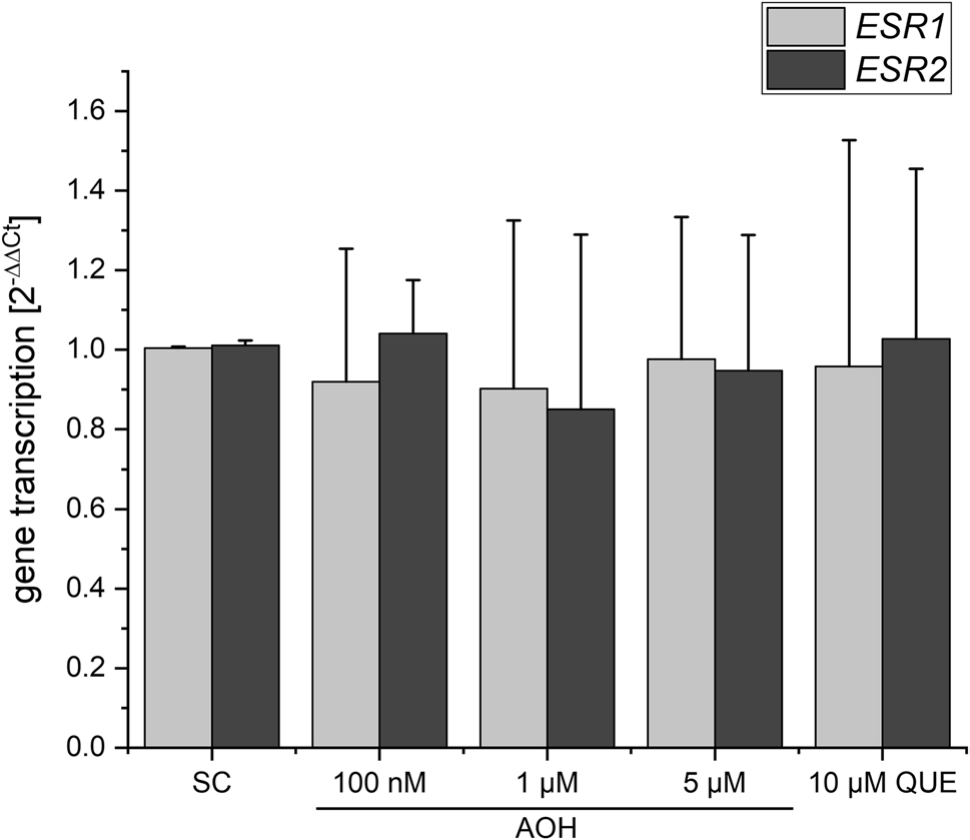
The influence of AOH on the transcription of ERα (ESR1) and ERβ (ESR2), as measured by qRT-PCR. Bars display transcript levels in relation to the solvent control (SC, 1% DMSO) and housekeeping genes as 2^−ΔΔCt^ values. Statistic testing revealed no significant differences

**Fig. 7:**
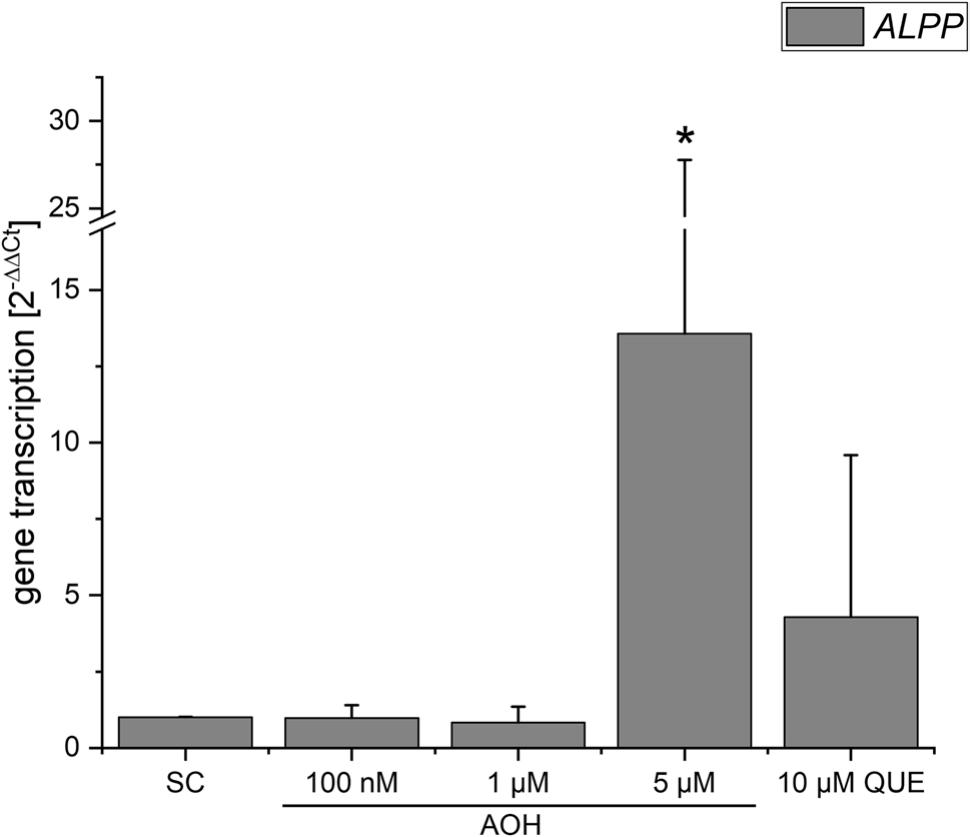
AlP (ALPP) transcription after a 24-h incubation with AOH, as measured by qRT-PCR. Bars display transcript levels in relation to the solvent control (SC, 1% DMSO) and housekeeping genes as 2^−ΔΔCt^ values. Significant differences to the solvent control were calculated by Mann–Whitney testing and are indicated by “*” (*p* < 0.05)

To assess whether the ability of AOH to inhibit CK2 might affect the transcription of estrogen receptors, we incubated estrogen-sensitive Ishikawa cells with the compound for 24 h and performed qRT-PCR measurements of *ESR1* and *ESR2* gene transcript levels (Fig. 6). Therewith, we could not observe any influence of AOH on those genes. Measuring after 5 h of incubation did not change this result (data not shown). Of note, also our positive control (10 µM QUE) did not alter ER gene transcription. Likewise, transcript levels of the *ALPP* gene, which is under the control of ERs and reflects estrogenicity, were determined after a 24-h incubation with AOH or the controls (Fig. 7). AOH significantly induced the transcription at the highest applied concentration (5 µM) by 13.5-fold in comparison to the solvent control. 10 µM QUE also showed some activity in that regard, which was however not statistically significant due to high deviation.

### ER localization

**Fig. 8:**
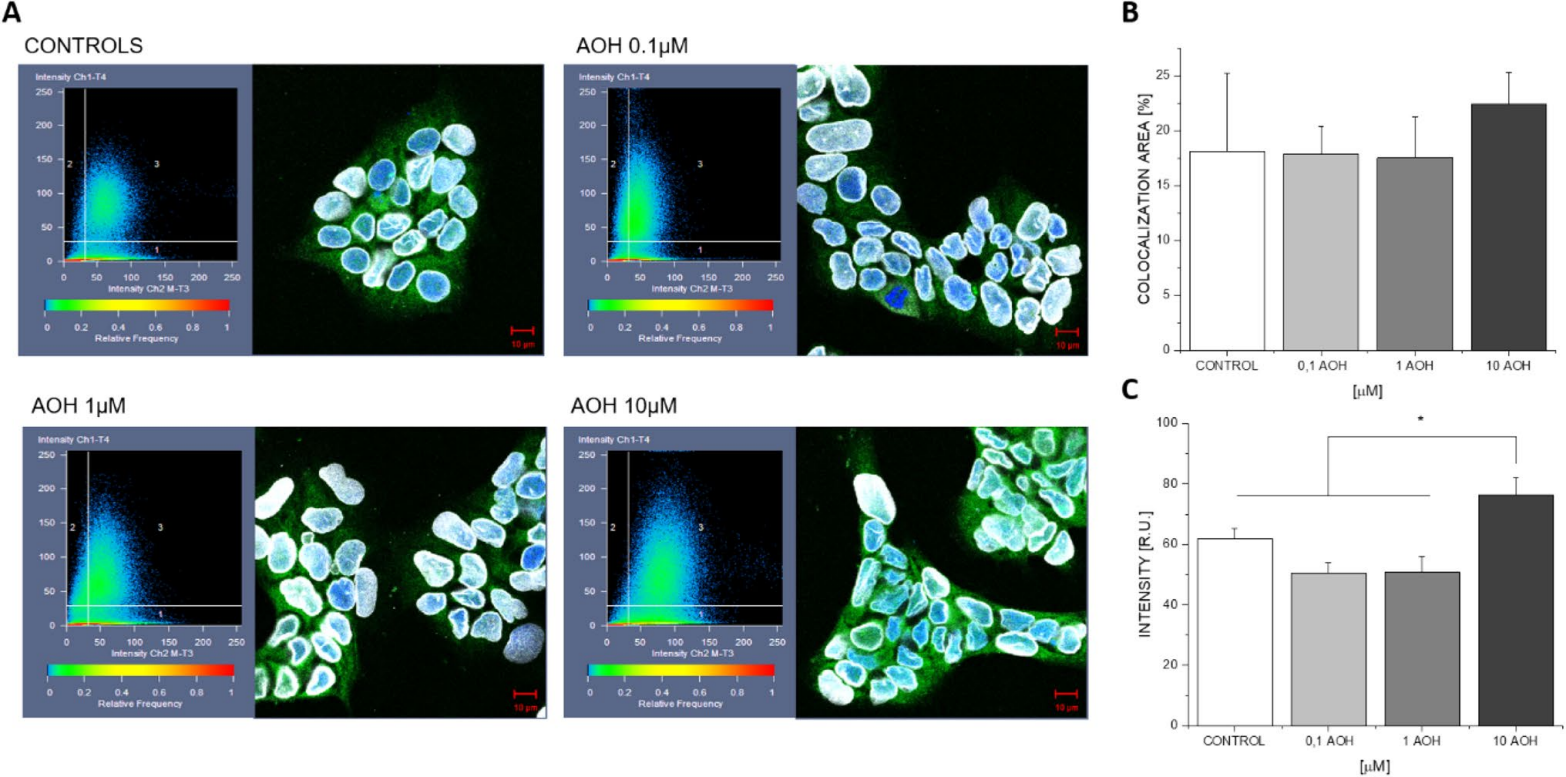
Localization of ERα, as measured by confocal microscopy. Representative images of Ishikawa cells incubated for 24 h with AOH or a respective solvent control, stained to detect DNA (blue) and ERα (green), with an included 2D graph displaying the frequency of co-occurring signals, indicating nuclear localization of the receptor. Quantification of co-localization between ERα and the DNA was carried out based on % AREA (**b**) and intensity relative units (**c**). Here, bars represent the mean + SD of at least four measurements. * indicates significant difference at Mann and Whitney test, *p* < 0.05 (color figure online)

In addition, we investigated possible effects on the localization of ERα in Ishikawa cells. In fact, the inhibition of CK2, which may impair ER phosphorylation, is expected to increase the DNA-binding affinity of ERα. To this aim, the intracellular localization of ERα was assessed by confocal microscopy. After 24 h of incubation with different concentrations of the mycotoxin, we observed an enhanced level of ERα in the nucleus at the highest applied concentration (10 µM), visible as a shift in the co-localization of ERα in the nuclear region (DAPI staining; Fig. 8a). Furthermore, when comparing the solvent control to 10 µM AOH, image analysis revealed a tendency towards an enhanced co-localization, as visible from the increase of signal intensity from 61.9 to 76.3 arbitrary units (Fig. 8c). However, it has to be pointed out that at 10 µM AOH is also expected to activate the ERα, promoting its translocation into the nucleus accordingly.

## Discussion

An in silico target fishing study relying on ligand-based virtual screening and structure-based molecular modeling was used to identify novel and unexpected targets of AOH, taken as the signature compound of *Alternaria* toxins. The ligand-based virtual screening revealed physico-chemical similarity between the mycotoxin AOH and well-described ATP-competitive inhibitors of CK2 such as 11-chloro-8-methyl-7H-benzo[e]pyrido[4,3-b]indol-3-ol, coumestrol and ellagic acid. In particular, the marked structural analogies with the natural polyphenol ellagic acid strongly supported the potential inhibitory activity of AOH. However, the possible existence of inhibitory activity was further confirmed by molecular modeling studies showing the capability of AOH to favorably dock the ATP pocket of CK2 and persist therein. In addition, to broaden the discovery of possible inhibitors among *Alternaria* mycotoxins, the capability of AME, ATX-II and altenuene to fit the ATP-binding site was assessed too. AME and ATX-II, but not altenuene, were found able to favorably interact. Notably, the predicted incapability of altenuene to compete with the ATP-binding site could not exclude possible enzyme inhibition via a different mechanism, given the multiple ways to inhibit CK2 (Prudent and Cochet 2009).

On the basis of in silico results, we tested AOH for the inhibition of that very enzyme using a cell-free in vitro assay. Therewith, AOH was confirmed to inhibit CK2 with an IC_50_ of 707 nM (Fig. 5). The tested concentrations should be considered to be of toxicological relevance, as plasma levels of the compound were previously reported to reach three-digit nanomolar concentrations during the first hour after an oral application in mice (Schuchardt et al. 2014).

CK2 is a target of increasing interest in pharmaceutical research. It has a vast array of physiological targets and participates in a complex series of cellular functions, including the maintenance of cell viability, the development of different malignancies (Gowda et al. 2017; Litchfield 2003), cell survival and DNA damage response (Rabalski et al. 2016). CK2 inhibitors mostly work by competing with ATP at the respective binding pocket (Gowda et al. 2017). Thus, based on the present study, alternariol might provide a promising scaffold for the development of novel CK2 inhibitors, but the result collected also raise the question on the relevance of CK2 for the toxicological mode of action of AOH.

We recently reported AOH to potentiate the estrogenic activity of several xenoestrogens at concentrations at which it did not exert estrogenic effects itself (Vejdovszky et al. 2017a, b). Thus, we addressed the question whether the apparent ability of the mycotoxin to inhibit CK2 might be responsible for that interaction. By conducting qRT-PCR measurements of ER transcript levels, and in line with our expectations based on previous results, we found AOH to induce the transcription of the *ALPP* gene (coding for the alkaline phosphatase protein) at a concentration of 5 µM (Fig. 7), but not at lower concentrations. This gene serves as a natural reporter for the induction of ER-dependent gene transcription in Ishikawa cells (Littlefield et al. 1990). However, we did not observe any influence of AOH or the well-documented CK2 inhibitor quercetin on the transcription of *ESR1* and *ESR2*, the genes coding for ERα and ERβ, respectively (Fig. 6). Of note, beyond CK2 inhibition, quercetin as a bioactive polyphenol has been associated with a multitude of potential cellular targets. Thus, an overlay of cellular mechanisms is always to be expected and compensatory effects are likely to occur. The same might have to be considered for AOH, which has already been demonstrated to affect several different cellular targets. Nevertheless, based on the present data, impact of AOH on the expression level of the estrogen receptors can be excluded as a potential sensitizing mechanisms involved in the abovementioned combinatory estrogenic effect. Considering the potential effect of CK2 on the translocation of ERs into the nucleus, confocal microscopy experiments were performed in Ishikawa cells showing an enhanced nuclear localization of ERα (24-h treatment at 10 µM; Fig. 8), which might reflect an enhanced binding to the DNA. These results are in good accordance with the observed enhanced transcription levels of the estrogen-sensitive *ALPP* gene (Fig. 7). It cannot be excluded that inhibition of CK2 is involved in an enhanced nuclear translocation of ERα. Nevertheless, multiple mechanisms may co-occur in explaining the abovementioned effect; at 10 µM AOH is expected to act as an ER agonist, thus promoting the nuclear localization of the protein. On this basis, an overlay of both mechanisms cannot be ruled out.

Furthermore, we assessed the potential of other co-occurring *Alternaria* toxins to inhibit CK2 with an in silico simulation of docking affinities to the ATP-binding pocket of the enzyme. The model predicted an interaction with the ATP pocket of CK2 not only for AOH, but also for the methylated form AME and for ATX-II. In particular, compounds of the perylene quinone family such as ATX-II are known for their strong genotoxic and mutagenic potential (Fleck et al. 2016; Schwarz et al. 2012). Thus, we decided to use ATX-II, isolated from an extract of *Alternaria alternata* cultured on rice as recently described (Puntscher et al. 2018a), to perform in vitro CK2 activity assays for a validation of the in silico prediction. In these experiments, ATX-II inhibited CK2 at concentrations ≥ 1 µM, with an IC_50_ of 5.1 µM (Fig. 5). Notably, AOH exerted a higher inhibitory effect in vitro, while the in silico approach predicted it to act weaker in comparison to ATX-II. This might arise from the well-known limited stability of ATX-II in aqueous solutions as previously reported (Aichinger et al. 2018). However, molecular dynamic simulations revealed the lower capability of ATX-II compared to AOH to persist within the ATP pocket over the time. Therefore, the lower geometrical stability of ATX-II-CK2, which is likely resulting in a ligand detachment earlier than that of AOH, could provide a mechanistic explanation of the experimental data collected. Concerning AME, the computational results collected pointed to a pattern of dynamic interactions with CK2 similar to those observed for ATX-II. On this basis, AME was predicted able to inhibit CK2 though its inhibitory potential was computed lower than that of AOH. Due to the already significant genotoxic impact at that concentration, the inhibition of kinases does not seem to be of high toxicological relevance for this particular substance. However, it can be seen as a proof of principle for our experimental approach and underlines the importance of testing other *Alternaria* toxins for CK2 inhibition in upcoming studies.

## Conclusion

We hereby report the reliability of our in silico target fishing approach in identifying novel and unexpected biological targets of low-molecular-weight molecules. Specifically, CK2 was described as a novel biological target of the mycotoxin alternariol. In the framework of the early analysis of mechanisms of toxicity, ATP-competitive inhibition of CK2 was pinpointed as a mechanism of possible concern deserving further characterization to define its relevance in the whole mechanism of action of AOH. ATX-II was also found able to inhibit CK2, but at higher concentrations than AOH. Furthermore, AME was also predicted to target CK2 (but with a lower computed inhibitory potential than AOH), pointing to possible combined effects when co-occurring with AOH, though its activity needs to be confirmed further in in vitro experiments.

## Abbreviations

AOH: Alternariol
AME: Alternariol monomethyl ether
ATX-II: Altertoxin II
CK2: Casein kinase 2
DAPI: 4′,6-Diamidino-2-phenylindole
E2: 17β-Estradiol
ER: Estrogen receptor
GOLD: Genetic Optimization for Ligand Docking
QUE: Quercetin
RMSD: Root-mean-square analysis
